# Supplementary material for: Discrete hippocampal projections are differentially regulated by parvalbumin and somatostatin interneurons
Source: Nat Commun. 2023 Oct 20;14:6653. doi: 10.1038/s41467-023-42484-z (PMC10589277; doi:10.1038/s41467-023-42484-z)
Supplement: Supplementary file 2 — Description of Additional Supplementary Files [file 41467_2023_42484_MOESM2_ESM.pdf]

Description of Additional Supplementary Files

File Name: Supplementary Data 1
Description: **Table of genes differentially expressed by vHipp projections to the mPFC and NAc.** Genes differentially expressed in mPFC- and NAc-projecting pyramidal cells. Data were analyzed by edgeR, which models count data using an overdispersed Poisson model, and uses an empirical Bayes procedure to moderate the degree of overdispersion across genes. $p<0.05$.

File Name: Supplementary Data 2
Description: **Differential gene expression between vHipp projections to mPFC and NAc cannot be explained by anatomical location.** Genes analyzed for differential expression between CA1 and subiculum by Cytosplore Viewer. Data were analyzed by the Wilcoxon rank-sum test and p values were corrected for multiple testing through Bonferroni correction. $p<0.05$.

File Name: Supplementary Data 3
Description: **Individual cell firing rates before and during optogenetic inhibition.** Firing rate of GFP-positive pyramidal cells before and during optogenetic inhibition of interneuron subtypes. Data were analyzed by two-way ANOVA, Laser $p=0.012$, Subject $p<0.0001$. Source data are provided as a Source Data file.

File Name: Supplementary Data 4
Description: **Flow cytometry gating strategy.** The gating strategy and cell counts for flow cytometry experiment is shown.
