# Supplementary figures and images for: Discrete hippocampal projections are differentially regulated by parvalbumin and somatostatin interneurons
Source: Nat Commun. 2023 Oct 20;14:6653. doi: 10.1038/s41467-023-42484-z (PMC10589277; doi:10.1038/s41467-023-42484-z)

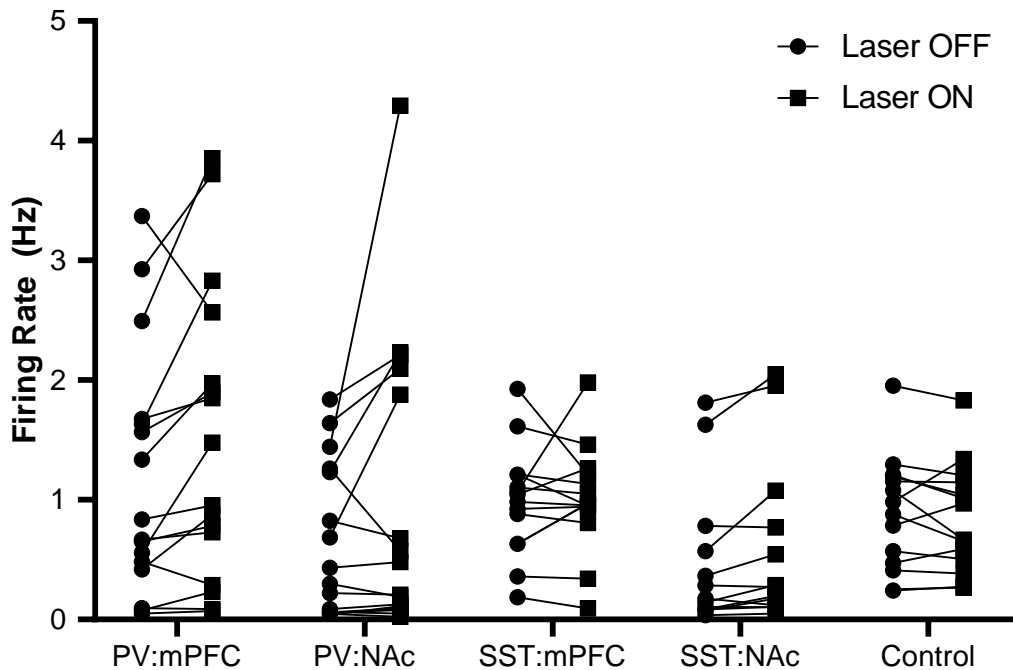

Supplement: Supplementary file 5 — Supplementary Data 3 [file 41467_2023_42484_MOESM5_ESM.pdf]
