# Supplementary material for: Discrete hippocampal projections are differentially regulated by parvalbumin and somatostatin interneurons
Source: Nat Commun. 2023 Oct 20;14:6653. doi: 10.1038/s41467-023-42484-z (PMC10589277; doi:10.1038/s41467-023-42484-z)
Supplement: Supplementary file 6 — Supplementary Data 4 [file 41467_2023_42484_MOESM6_ESM.pdf]

Batch Analysis Report

Run Date: 4/10/20 4:48 PM

Experiment: 2020-04-10 RFP MS BRAIN

User ID: Lodge

Statistics Output: N/A

Worksheet PDF Output: Z:\Users\Lodge\Aria\2020\2020-04-10 RFP MS BRAIN\2020-04-10 RFP MS BRAIN.pdf

Specimen_001

Tube	Status	Run Time
CONTROL	OK	4/10/20 4:48 PM
P1	OK	4/10/20 4:49 PM
P2	OK	4/10/20 4:49 PM
P3	OK	4/10/20 4:49 PM
P4	OK	4/10/20 4:49 PM
P5	OK	4/10/20 4:49 PM
N1	OK	4/10/20 4:49 PM
N2	OK	4/10/20 4:50 PM
N3	OK	4/10/20 4:50 PM
N4	OK	4/10/20 4:50 PM
N5	OK	4/10/20 4:50 PM
N6	OK	4/10/20 4:51 PM

BD FACSDiva 8.0.1

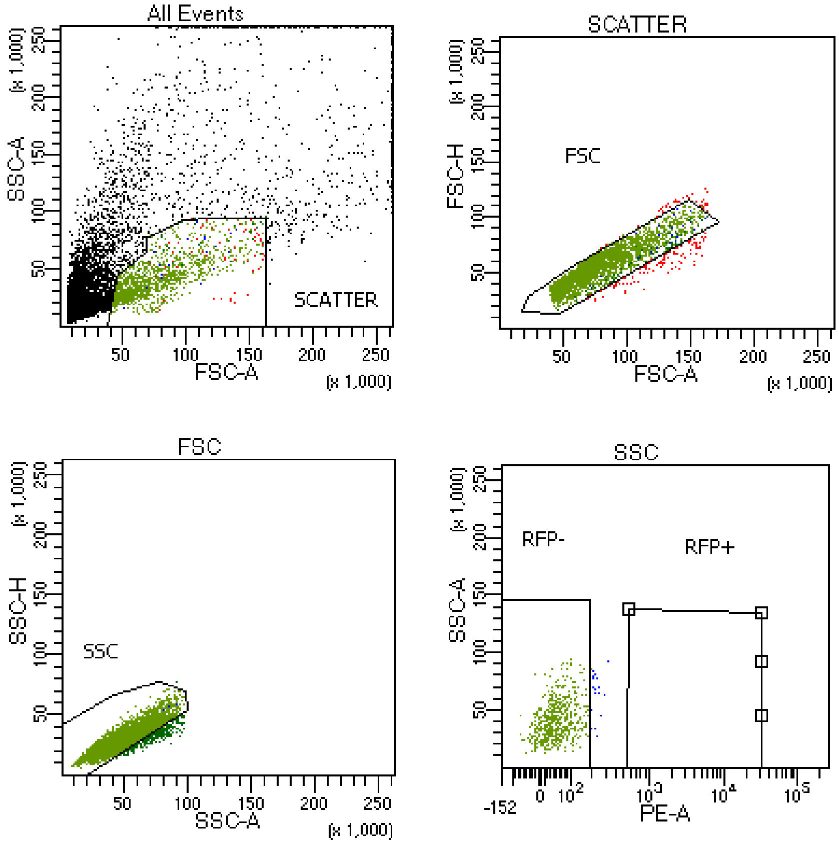

Tube: CONTROL

Population	#Events	%Parent	%Total
All Events	50,000	####	100.0
SCATTER	6,602	13.2	13.2
FSC	6,268	94.9	12.5
SSC	5,955	95.0	11.9
RFP+	0	0.0	0.0
RFP-	5,812	97.6	11.6

BD FACSDiva 8.0.1

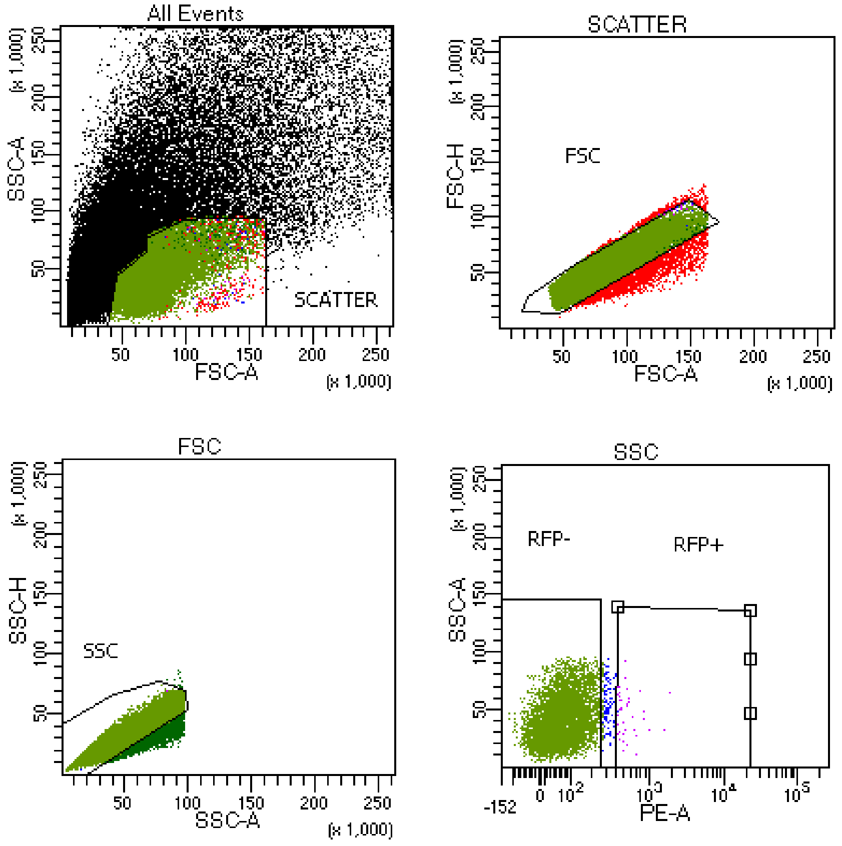

Tube: P1			
Population	#Events	%Parent	%Total
All Events	694,540	####	100.0
SCATTER	79,694	11.5	11.5
FSC	73,519	92.3	10.6
SSC	67,796	92.2	9.8
RFP+	426	0.6	0.1
RFP-	66,365	97.9	9.6

BD FACSDiva 8.0.1

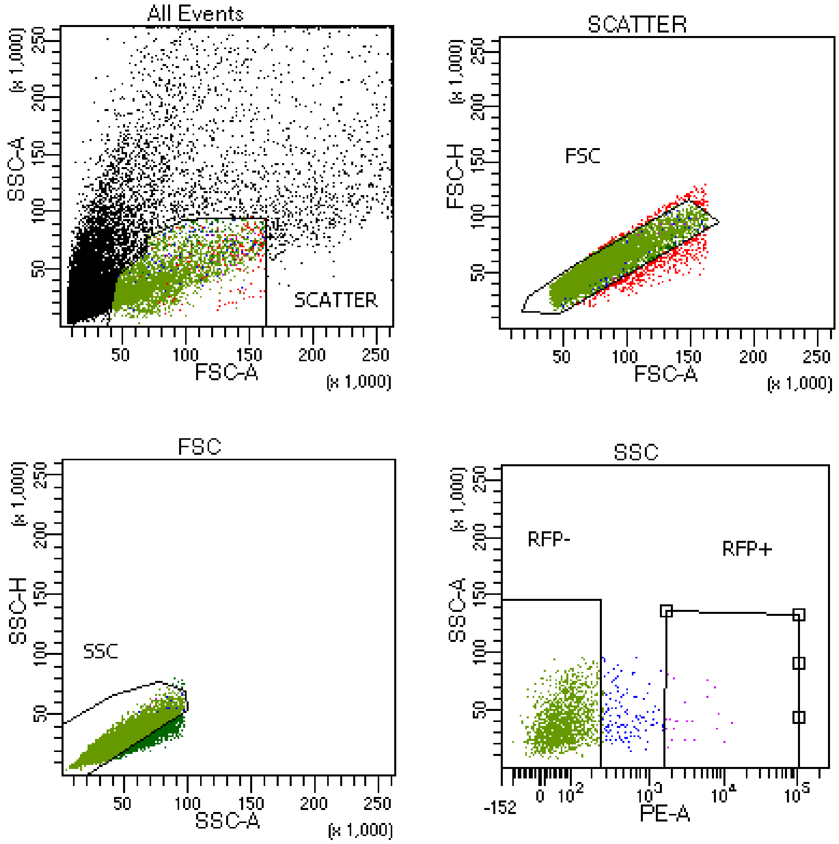

Tube: P2			
Population	#Events	%Parent	%Total
All Events	138,149	####	100.0
SCATTER	17,999	13.0	13.0
FSC	16,776	93.2	12.1
SSC	15,925	94.9	11.5
RFP+	173	1.1	0.1
RFP-	14,682	92.2	10.6

BD FACSDiva 8.0.1

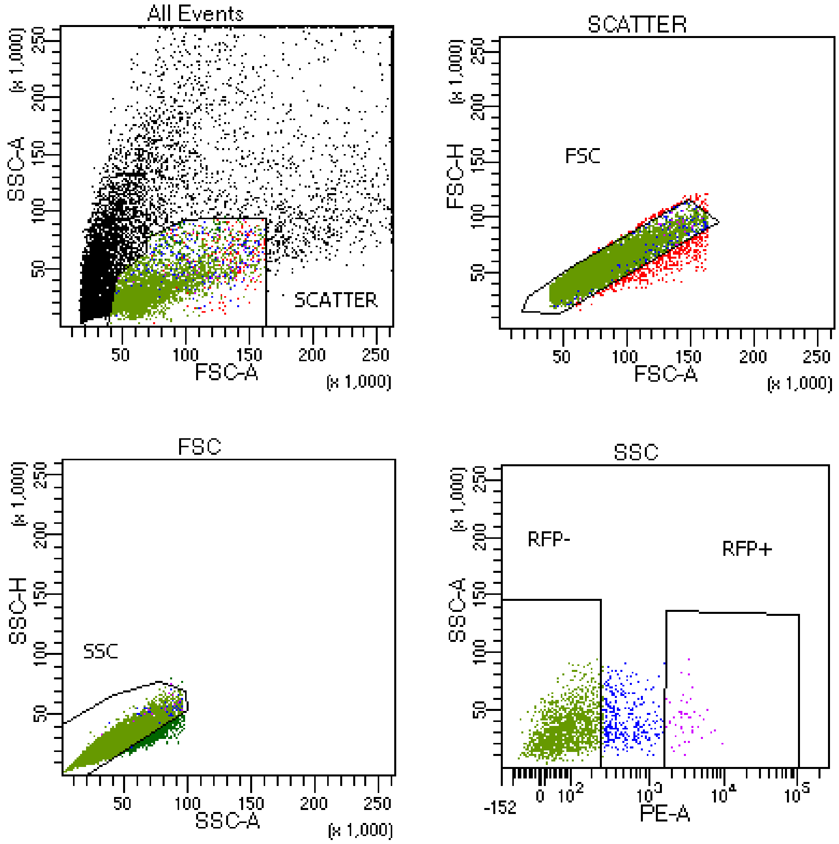

Tube: P3

Population	#Events	%Parent	%Total
All Events	72,824	####	100.0
SCATTER	20,442	28.1	28.1
FSC	19,266	94.2	26.5
SSC	18,687	97.0	25.7
RFP+	438	2.3	0.6
RFP-	15,295	81.8	21.0

BD FACSDiva 8.0.1

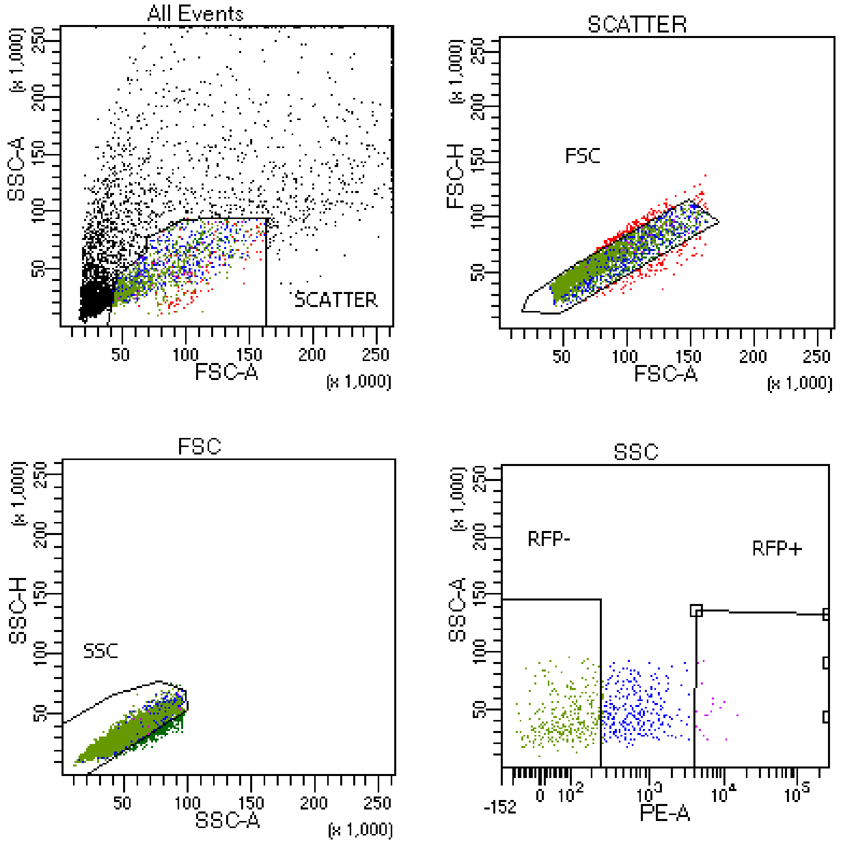

Tube: P4

Population	#Events	%Parent	%Total
All Events	27,033	####	100.0
SCATTER	7,098	26.3	26.3
FSC	6,530	92.0	24.2
SSC	6,180	94.6	22.9
RFP+	126	2.0	0.5
RFP-	3,296	53.3	12.2

BD FACSDiva 8.0.1

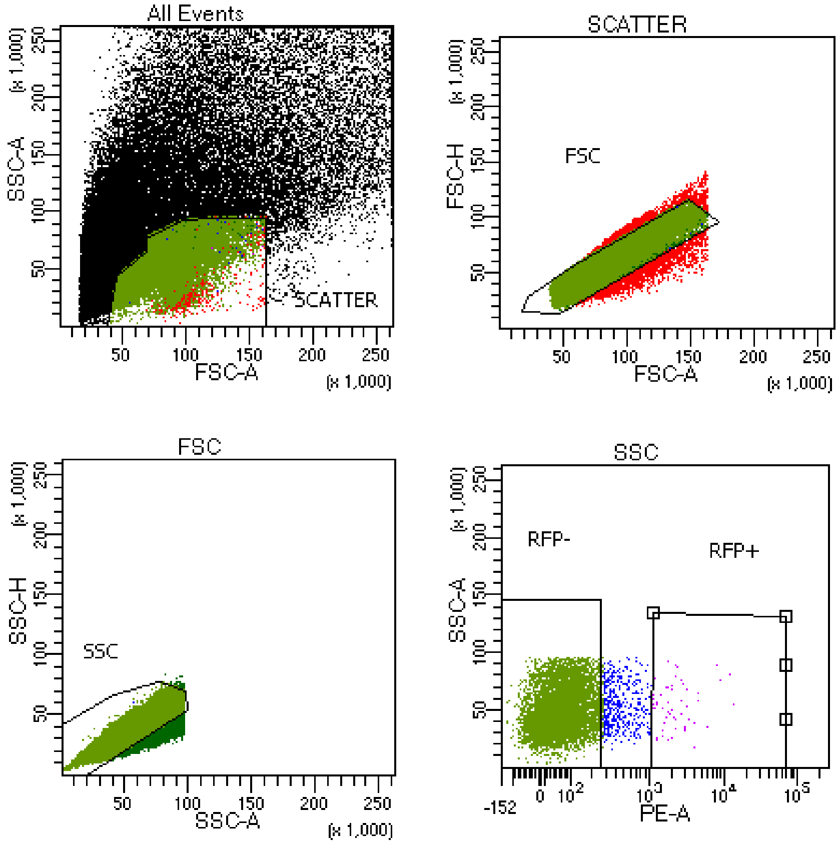

Tube: P5

Population	#Events	%Parent	%Total
All Events	493,545	####	100.0
SCATTER	96,768	19.6	19.6
FSC	86,727	89.6	17.6
SSC	80,331	92.6	16.3
RFP+	502	0.6	0.1
RFP-	75,626	94.1	15.3

BD FACSDiva 8.0.1

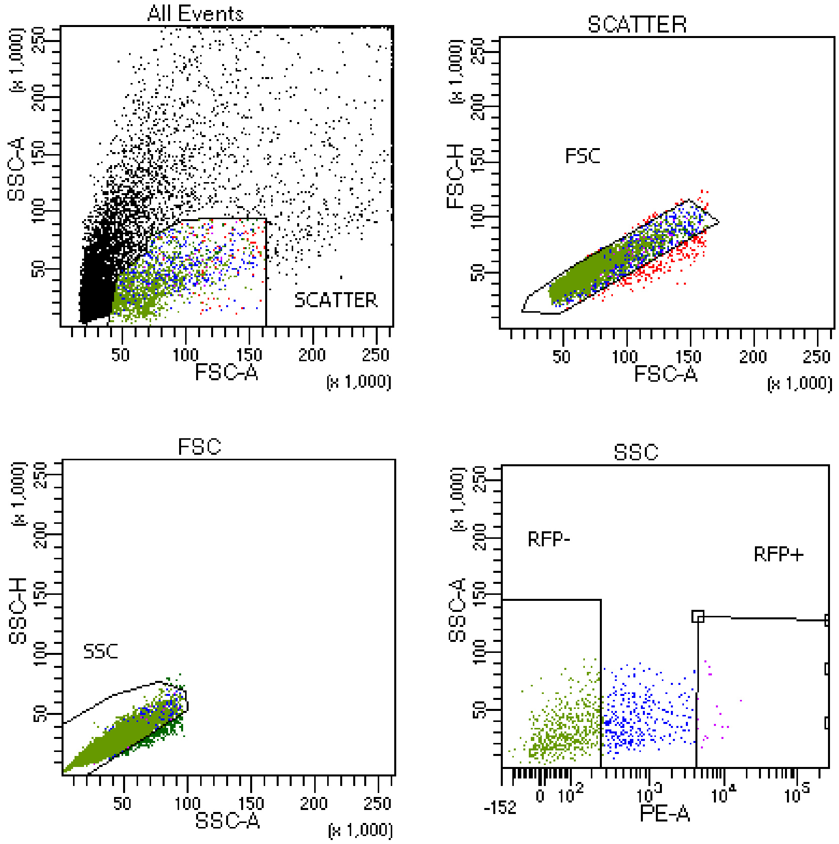

Tube: N1			
Population	#Events	%Parent	%Total
All Events	47,334	####	100.0
SCATTER	10,236	21.6	21.6
FSC	9,678	94.5	20.4
SSC	9,310	96.2	19.7
RFP+	243	2.6	0.5
RFP-	6,057	65.1	12.8

BD FACSDiva 8.0.1

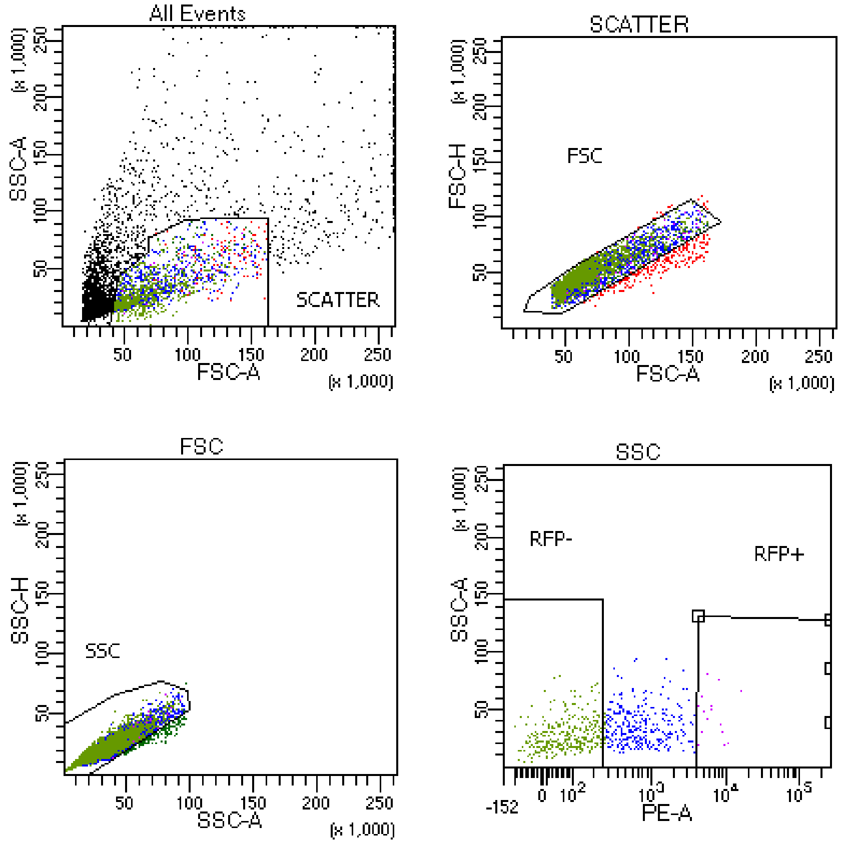

Tube: N2			
Population	#Events	%Parent	%Total
All Events	21,652	####	100.0
SCATTER	7,315	33.8	33.8
FSC	6,771	92.6	31.3
SSC	6,553	96.8	30.3
RFP+	180	2.7	0.8
RFP-	3,310	50.5	15.3

BD FACSDiva 8.0.1

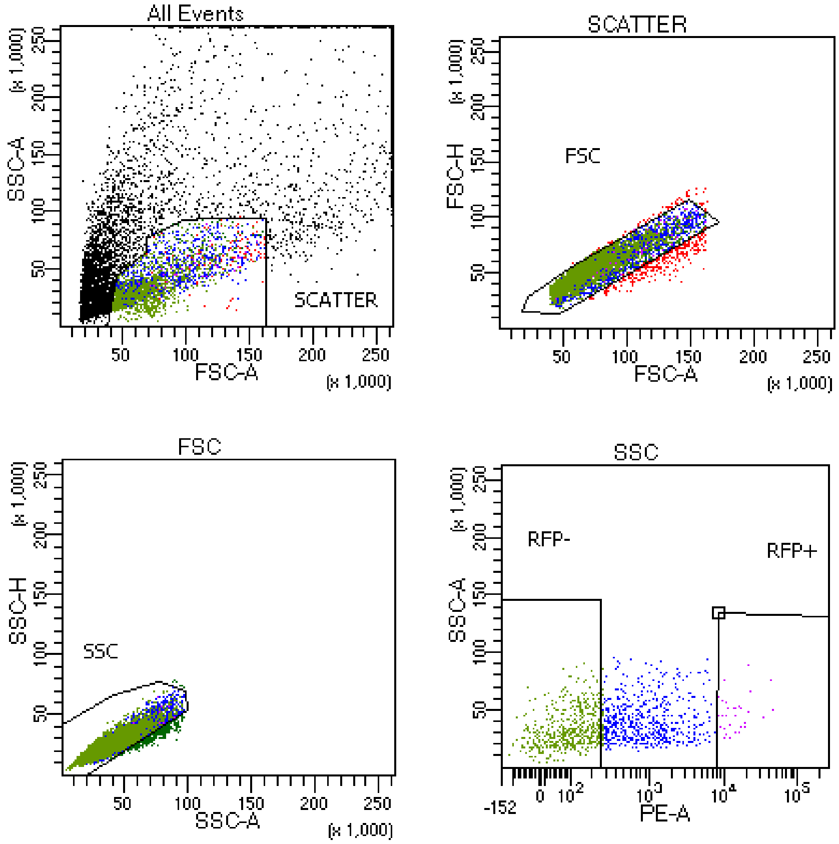

Tube: N3

Population	#Events	%Parent	%Total
All Events	39,975	####	100.0
SCATTER	11,920	29.8	29.8
FSC	11,187	93.9	28.0
SSC	10,802	96.6	27.0
RFP+	239	2.2	0.6
RFP-	5,197	48.1	13.0

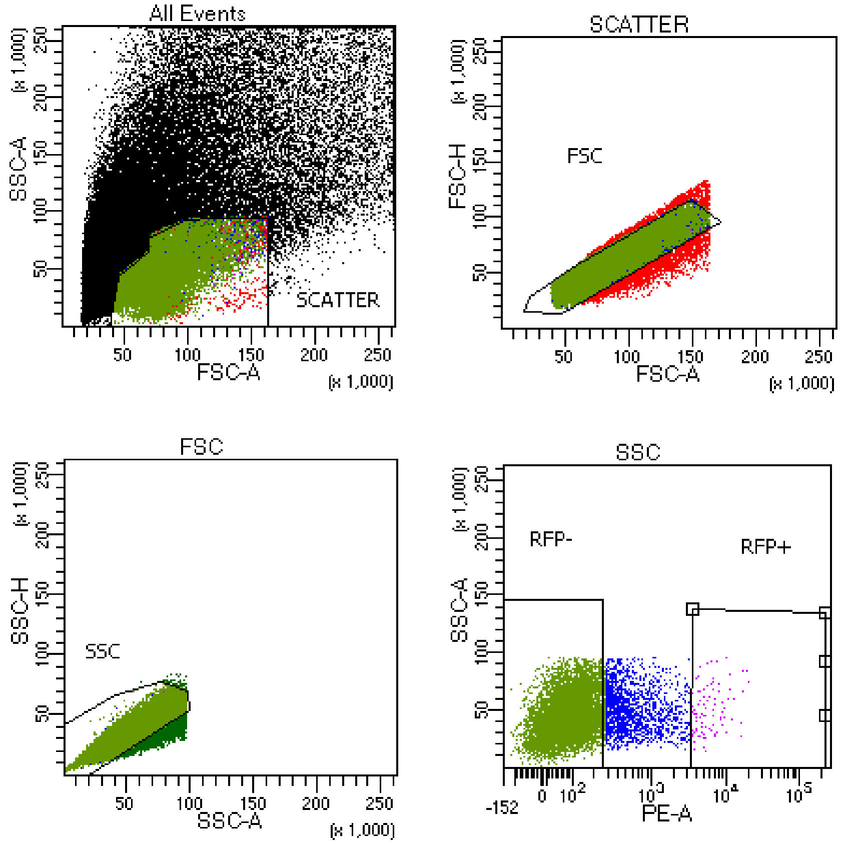

Tube: N4

Population	#Events	%Parent	%Total
All Events	498,355	####	100.0
SCATTER	100,306	20.1	20.1
FSC	91,263	91.0	18.3
SSC	85,515	93.7	17.2
RFP+	820	1.0	0.2
RFP-	70,036	81.9	14.1

BD FACSDiva 8.0.1

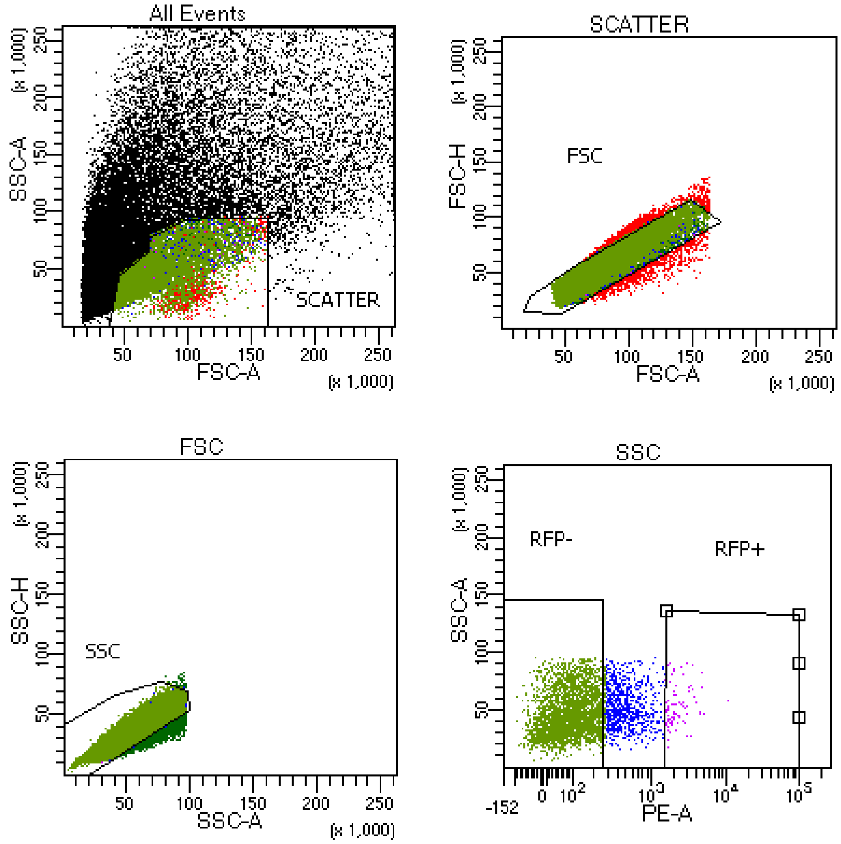

Tube: N5

Population	#Events	%Parent	%Total
All Events	242,991	####	100.0
SCATTER	48,322	19.9	19.9
FSC	43,168	89.3	17.8
SSC	40,429	93.7	16.6
RFP+	615	1.5	0.3
RFP-	32,486	80.4	13.4

BD FACSDiva 8.0.1

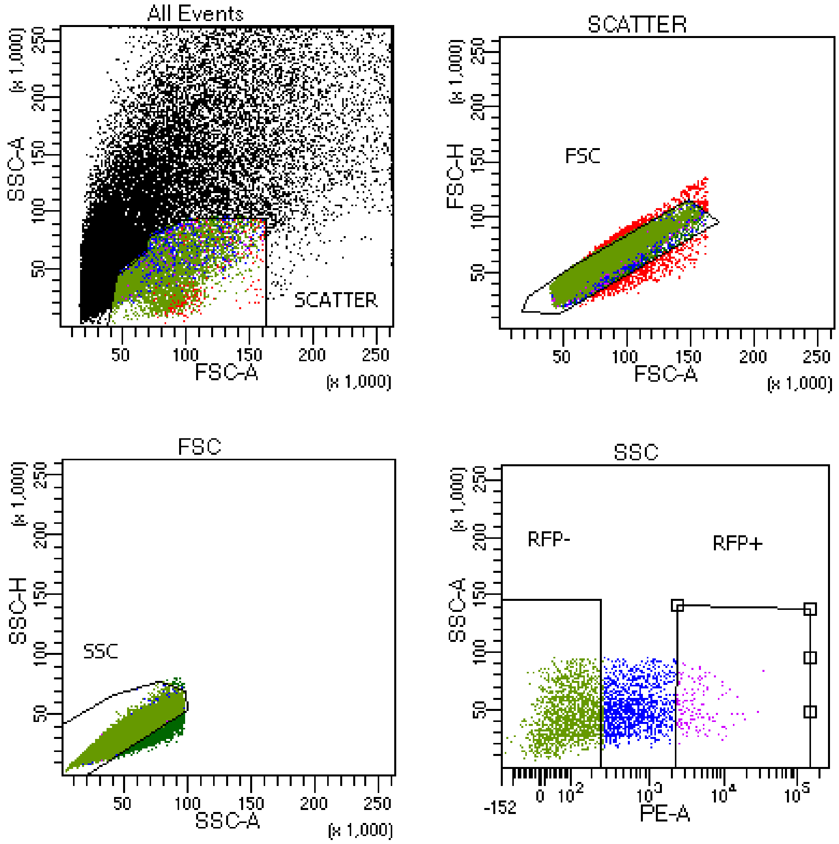

Tube: N6

Population	#Events	%Parent	%Total
All Events	211,900	####	100.0
SCATTER	33,842	16.0	16.0
FSC	29,716	87.8	14.0
SSC	27,555	92.7	13.0
RFP+	1,348	4.9	0.6
RFP-	14,764	53.6	7.0
